# Azacytidine induces necrosis of multiple myeloma cells through oxidative stress

**DOI:** 10.1186/1477-5956-11-24

**Published:** 2013-06-13

**Authors:** Enbing Tian, Haiping Tang, Renhua Xu, Chongdong Liu, Haiteng Deng, Qingtao Wang

**Affiliations:** 1 Beijing Chaoyang Hospital affiliated Capital Medical University, Beijing, China; 2 School of Life Sciences, Tsinghua University, Beijing, China

**Keywords:** Necrosis, Cell-bound albumin, Proteomics, Heat shock proteins, Oxidative stress, Myeloma cells

## Abstract

Azacytidine is an inhibitor of DNA methyltransferase and is known to be an anti-leukemic agent to induce cancer cell apoptosis. In the present study, multiple myeloma cells were treated with azacytidine at clinically relevant concentrations to induce necrosis through oxidative stress. Necrotic myeloma cells exhibit unique characteristics, including enrichment of the cell-bound albumin and overexpression of endoplasmic reticulum (ER)- and mitochondrial-specific chaperones, which were not observed in other necrotic cells, including HUH-7, A2780, A549, and Hoc1a. Proteomic analysis shows that HSP60 is the most abundant up-regulated mitochondrial specific chaperone, and azacytidine-induced overexpression of HSP60 is confirmed by western blot analysis. In contrast, expression levels of cytosolic chaperones such as HSP90 and HSP71 were down-regulated in azacytidine-treated myeloma cells, concomitant with an increase of these chaperones in the cell culture medium, suggesting that mitochondrial chaperones and cytosolic chaperones behave differently in necrotic myeloma cells; ER- and mitochondrial-chaperones being retained, and cytosolic chaperones being released into the cell culture medium through the ruptured cell membrane. Our data suggest that HSP60 is potentially a new target for multiple myeloma chemotherapy.

## Background

Multiple myeloma (MM) is a clonal B-cell disorder in which malignant plasma cells (PC) accumulate in the bone marrow, resulting in lytic bone lesions and excessive amounts of monoclonal proteins. It accounts for 10% of hematologic malignancies. Although therapeutic interventions have been developed and the overall survival has been improved over the last decade [1], myeloma is still incurable and most multiple myeloma patients who survive initial treatment will develop drug resistance, and eventually relapse. Development of new therapeutic interventions is strikingly needed for increasing patient survival rate. It has been shown that abnormal methylation of tumor suppressor genes is a common event in malignant plasma cell disorders [2-4] and aberrant global methylation patterns also affect the molecular pathogenesis of myeloma [3]. Azacytidine, a ring analog of the naturally occurring pyrimidine nucleoside cytidine, is an inhibitor of DNA methyltransferase. Following incorporation into DNA, azacytidine is capable of covalently binding to DNA methyltransferase, resulting in hypomethylation and transcriptional reactivation of some silenced genes. This has led to development of azacytidine as a therapeutic anti-cancer agent. Azacytidine has been used to treat patients with myelodysplastic syndrome and acute myeloid leukemia at the dose of 75-100 mg/day [5-7]. In vitro studies have shown that azacytidine alone or in combination with other antitumor agents induces tumor cell apoptosis. It has been reported that azacytidine induced ATR-mediated DNA double-strand break responses, apoptosis, and synergistic cytotoxicity in multiple myeloma cells [8]. It has also been demonstrated that a combination of azacytidine and arsenic trioxide generates a synergistic anti-tumor activity in myeloma [9]. Moreover, azacytidine has been shown to activate the interleukin-6 and nuclear factor-kB signaling pathways [10], and to induce overexpression of semenogelin I in myeloma cells [11]. To the best of our knowledge, azacytidine induced necrosis in myeloma cells has not yet been reported.

Necrosis is one type of cell death that lacks characteristics of apoptosis and autophagy [12-16]. Over the last several years, the occurrence and course of necrosis was found to be programmed and tightly regulated. Extensive studies show that death ligands (*e.g.,* CD95L, TNF and TNF-related apoptosis-inducing ligand) induce caspase-independent necrotic-like cell death that relies on the activity of the death domain (DD)-containing kinase RIP1. Although the induction mechanisms of necrosis are becoming increasingly clear, the execution of this process remains somewhat elusive. Necrosis is accompanied by a complex sequence of cellular processes including mitochondrial dysfunction with enhanced generation of reactive oxygen species (ROS) and ATP depletion, proteolysis by calpains and cathepsins, and early plasma membrane rupture. One important consequence of necrosis is the induction of immunogenic responses pursuant to the release of immunogens from necrotic cells [17-20]. *Basu* and colleagues reported that heat shock proteins (HSPs) including gp96, calreticulin, HSP90 and HSP72 were released into the culture supernatant from necrotic cells in response to freeze thaw, but not from apoptotic cells [21,22]. It was further shown that the released HSPs activated the NF-κB pathway, stimulated macrophages to secrete cytokines, induced the expression of co-stimulatory molecules, and enhanced antigen presentation in dendritic cells [23-28].

A few studies have been reported on necrosis of myeloma cells. Kigamicin, a compound derived from actinomycetes, induces necrosis in human myeloma cells by inhibition of cyclin D1, p21, p-AKT, and p-ERK [29]. A D-amino acid-containing peptide HYD1 increases the reactive oxygen species production, leading to necrotic cell death in multiple myeloma cells [30]. When cells are treated with azacytidine, not only DNA methyltransferase are inhibited, but ROS generation is also observed [31]. For example, ROS generation is used as an indicator for the synergistic and cytotoxic effects of azacytidine in AML and acute lymphoblastic leukemia cells [32,33]. Within cells, mitochondria are susceptible targets for oxidant stress. ROS can modify mitochondrial lipids, proteins, and DNA. The lack of histones in mtDNA also makes mitochondria more vulnerable to oxidative stress [34,35]. Oxidative stress also may lead to modifications and alterations of endoplasmic reticulum (ER) chaperone proteins [36], causing the accumulation of unfolded or misfolded proteins, and decreases in protein synthesis. In the present work, we show that azacytidine-treatment induces necrosis of myeloma cells through oxidative stress, and that necrotic myeloma cells exhibit unique characteristics, including enrichment of cell-bound albumin and overexpression of the ER- and mitochondrial-specific chaperones. Expression of HSP60 has been shown to exhibit the largest increase upon azacytidine treatment and HSP60 is a potential binding partner of cell-bound albumin.

## Methods

### Chemicals and reagents

RPMI1640 medium, phosphate-buffered saline (PBS) and fetal bovine serum were purchased from Wisent (Montreal, QC) and used without further purification. Dithiothreitol (DTT) was purchased from Merck (Whitehouse Station, NJ). Sequencing grade modified trypsin was purchased from Promega (Fitchburg, WI). 5-azacytidine, iodoacetamide (IAA) and RNase A were purchased from Sigma (St Louis, MO). Dimethyl sulfoxide was purchased from Applichem (St Louis, MO). A BCA protein assay kit was purchased from Solarbio (Tongzhou District, Beijing). TMT^®^ Mass Tagging Kits and Reagents were purchased from Thermo Scientific (Rockford, IL).

### Cell Culture and Sample Preparation

Human MM cell line U266 was purchased from the Tumor Cell Bank of Chinese Academy of Medical Sciences (Beijing, China), and NCI-H929 and RPMI-8226 cells were kindly provided by Dr. Wenming Chen (Beijing Chao-Yang Hospital Affiliated to the Capital University of Medical Science). All three cell lines were cultured in RPMI 1640 (Wisent) containing 10% or 15% fetal bovine serum with 100 units/mL penicillin and 100 μg/mL streptomycin at 37°C in a humidified incubator with 5% CO_2_. Prior to treatment, cells were cultured for at least 12 h to reach exponential growth phase. Cells were treated with azacytidine dissolved in dimethyl sulfoxide and the control cells were treated with the same amount of DMSO for the same time periods. After treatments, cells were washed twice with ice-cold PBS and lysed with RIPA lysis buffer (25 mmol/L Tris-HCl pH 7.6, 150 mmol/L NaCl, 0.1% SDS, 1% NP-40, 1% sodium deoxycholate, 1 mmol/L PMSF, and Roche Complete Protease Inhibitor Cocktail) for 30 min on ice. Cell lysates were clarified by centrifugation at 14, 000 ×g for 20 min at 4°C. The protein concentration in the supernatant of each sample was determined using a BCA protein assay kit.

### Protein Separation by 1D SDS-PAGE and Proteomics Analysis

Equal amount of proteins from untreated- and azacytidine-treated samples (about 30 μg) were separated by 1D SDS-PAGE, respectively. The gel bands of interest were excised from the gel, reduced with 25 mM of DTT and alkylated with 55 mM iodoacetamide. In gel digestion was then carried out with sequencing grade modified trypsin in 50 mM ammonium bicarbonate at 37°C overnight. Peptides were extracted twice with 0.1% trifluoroacetic acid in 50% acetonitrile aqueous solution for 30 min. The extracts were then centrifuged in a speedvac to reduce the volume. To analyze the proteins in the cell culture medium of untreated and azacytidine-treated U266 cells, cells were cultured in RPMI 1640 medium containing 0.5% fetal bovine serum for 48 h and the culture mediums were collected. Proteins from the same volume of cell culture mediums were separated by 1D SDS-PAGE, and the gel bands were excised and digested with trypsin. For protein quantitation, peptides from different samples were labeled with TMT reagents purchased from Thermo-Pierce Biotechnology (Rockford, IL) according to the manufacturer’s instructions. Briefly, TMT reagents were dissolved in anhydrous acetonitrile. Labeling reaction was carried out by incubation of tryptic peptides with the TMT reagents for 1 h at room temperature, and the reaction was quenched with hydroxylamine. TMT-labeled peptides were desalted using the stage tips.

For LC-MS/MS analysis, the digestion product was separated by a 65 min gradient elution at a flow rate 0.250 μL/min with an EASY-nLCII™ integrated nano-HPLC system (Proxeon, Denmark) which was directly interfaced with a Thermo LTQ-Orbitrap mass spectrometer. The analytical column was a home-made fused silica capillary column (75 μm ID, 150 mm length; Upchurch, Oak Harbor, WA) packed with C-18 resin (300 Å, 5 μm, Varian, Lexington, MA). Mobile phase A consisted of 0.1% formic acid, and mobile phase B consisted of 100% acetonitrile and 0.1% formic acid. The LTQ-Orbitrap mass spectrometer was operated in the data-dependent acquisition mode using Xcalibur 2.0.7 software and there was a single full-scan mass spectrum in the Orbitrap (*m/z* 400 to *m/z* 1800, 30,000 resolution) followed by 20 data-dependent MS/MS scans in the ion trap at 35% normalized collision energy (CID). The MS/MS spectra from each LC-MS/MS run were searched against the selected database using an in-house Proteome Discovery searching algorithm. For quantitation by TMT labeling, TMT-labeled peptides were analyzed by nano-LC-MS/MS with a Q Exactive mass spectrometer that was also operated in the data-dependent acquisition mode using the Xcalibur 2.1.2 software and there was a single full-scan mass spectrum in an Orbitrap (m/z 350 to m/z 1600 Da) followed by 10 MS/MS scans. The MS/MS spectra from each LC-MS/MS run were analyzed using Proteome Discoverer (Version 1.3) for protein identification and quantitation. Intensity ratios of the TMT reporter ions were used to determine relative concentrations of labeled proteins.

The search criteria were as follows: full tryptic specificity was required; one missed cleavages was allowed; carbamidomethylation was set as fixed modification; oxidation (M) was set as variable modification; precursor ion mass tolerances were set at 10 ppm for all MS acquired in the Orbitrap mass analyzer; and the fragment ion mass tolerance was set at 0.8 Da for all MS/MS spectra acquired in the linear ion trap. A proteins was designated as a “hit” only when 2 or more unique peptides with high confidence scores (FDR < 1%) were identified and their corresponding MS/MS spectra were manually inspected. When several proteins matched the same sets of peptides, only the proteins with the greater percentage of coverage was selected. Significance was regarded only when the ratio of spectral counts between two groups were more than 2 or less than 0.5.

### DNA Fragment Assay

DNA fragment assay was performed following the procedure described by Mazars et al [37]. Briefly, cells were washed with PBS twice and collected by centrifugation. Cells were suspended in 250 μl lysis buffer (1% NP-40, 20 mM EDTA, 50 mM Tris-HCl, pH 7.5). The supernatants were collected by centrifugation for 5 min at 1,600 × g. The supernatant was incubated with 0.71 mg/ml RNase A for 2 h at 56°C. Then 100 μg/ml pronase E was added and incubated with the supernatants overnight at 37°C. DNA fragments were precipitated with 0.5 volumes of 10 M ammonium acetate and 2 volumes of ethanol at -20°C for 12 h and centrifugated for 15 min at 15,000 ×g. The precipitate was washed with 70% ethanol and resuspended in loading buffer. Electrophoresis was performed in 0.5 × Tris-borate-EDTA buffer for 30 min.

### Flow Cytometry

Cells were spun down at 1000 ×g for 5 min. The medium was discarded and washed with PBS twice. Cells were resuspended in 75% ethanol, vortexed to mix briefly, and fixed at 4°C overnight. After vortex, the fixed cells were re-suspended with PBS solution containing Propidium Iodide (PI) (50 μg/ml), followed by RNaseA treatment (1 mg/ml) for 30 min at 37°C and analyzed with a BD FACSCalibur™ Flow Cytometer using 488 nm excitation and a 515 nm bandpass filter for fluorescein detection and a filter >560 nm for PI detection. Dot plots and histograms were analyzed by CellQuest Pro software (BD Biosciences, Heidelberg, Germany).

### Western Blot Analysis

Untreated- and azacytidine treated-cells were collected and lysed on ice with Biyuntian cell lysis buffer containing 20 mM Tris (pH 7.5), 150 mM NaCl, 1% Triton X-100, and sodium pyrophosphate, ß-glycerophosphate, EDTA, and Na_3_VO_4_ for Western and IP supplied with the protease inhibitor cocktail. The supernatants were collected after centrifugation at 14,000 ×g for 10 min at 4°C. Protein concentrations were determined using the BCA protein assay kit. Proteins were separated on a 12% SDS-PAGE gel and transferred onto a PVDF transfer membrane by electroblotting. After blocking with 5% nonfat milk for 2 h at room temperature, the membrane was incubated overnight at 4°C with 1000× diluted primary antibody, washed with PBST buffer for 3 times, then incubated with 1000× diluted anti-mouse or anti-rabbit secondary antibody labeled with HRP at room temperature for 2 h. The membrane was further washed with PBST buffer 3 times and developed using the Enlight Kit (Engreen, China). Glyceraldehyde 3-phosphate dehydrogenase (GAPDH) was detected with anti- GAPDH antibody as an internal control.

### Quantitative real-time PCR (qPCR)

Cells were harvested after being treated with azacytidine for different periods of time. Total RNA was extracted by the SV Total RNA Isolation System. cDNA was synthesized from 4 μg total RNA using the GoScript^TM^ Reverse Transcription System. All qPCR was performed using the Roche LightCycler® 480II Detection System with SYBR green incorporation according to the manufacturer’s instructions. The primers were either designed by using the Primer Premier 5 software or from Primer Bank (http://pga.mgh.harvard.edu/primerbank/). To prevent amplification of genomic DNA, all target primers span exon-exon junctions. The specific PCR products were confirmed by melting curve analysis. Relative expression was analyzed using the 2^-ΔΔCt^ method. Primer sequences for qPCR are listed in Additional file 1: Table S1.

### Co-immunoprecipitation

Cells were lysed with Biyuntian cell lysis buffer for Western and IP supplied with the protease inhibitor cocktail. The protein concentrations of the cell lysates were determined using a BCA assay. The protein A/G agarose beads were washed three times with cell lysis buffer. 2 mg of the cell lysate was incubated with 6 μg anti-bovine albumin antibody and 20 μl protein A/G agarose beads overnight at 4°C. The beads were centrifuged at 1000 ×g for 1 min and washed three times with the cell lysis buffer, and proteins bound to the Protein A/G agarose beads were then eluted by boiling the beads for 5 min in 1 × SDS loading buffer. Each eluent was separated by 1D SDS-PAGE followed by western blot analysis.

### Detection of Reactive Oxygen Species (ROS) in Untreated and Azacytidine-treated Cells

ROS in untreated and azacytidine-treated cells was detected using an Image-iT™ LIVE Green Reactive Oxygen Species Detection Kit (Molecular Probes, Inc. Eugene, OR) following the manufacturer’s instructions. Briefly, the cells were collected by centrifugation and washed once with warm HBSS/Ca/Mg. Cells were re-suspended with 500 μl of the 25 μM carboxy-H_2_DCFDA working solution for 25 min at 37°C, followed by addition of the Hoechst 33342 reagent to the reaction mixture at a final concentration of 1.0 μM and incubation for 5 min. The final products were washed gently with 1 ml HBSS/Ca/Mg immediately followed by imaging with Zeiss 710 Confocal Microscopy.

## Results and discussion

### Azacytidine induces myeloma cell necrosis through oxidative stress

**Figure 1:**
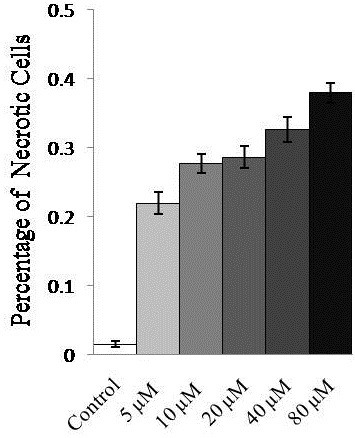
**Percentage of necrosis-related cell death in U266 cells treated with azacytidine (0–80 μM) for 24 h.** Results are expressed as the mean of three experiments. Significant necrosis was observed with 5 μM azacytidine treatment.

**Figure 2:**
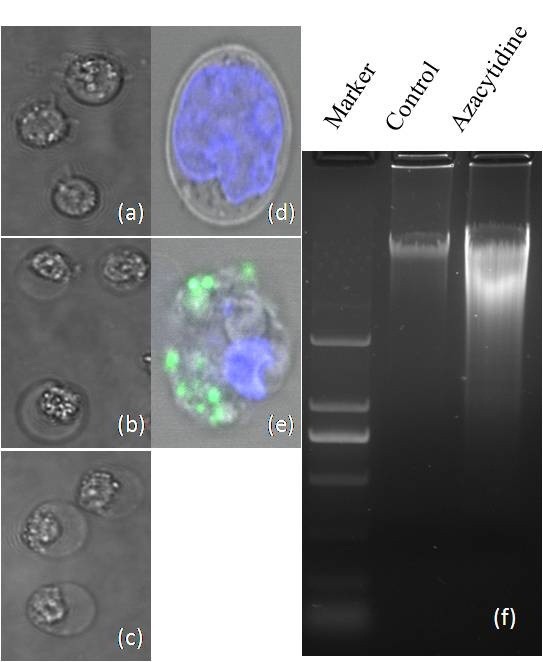
**Morphologic images of myeloma cells. (a)** untreated cells; **(b)** 100 μM azacytidine-treated cells for 24 h; and **(c)** H_2_O_2_-treated U266 cells; All images were captured by Olympus IX2-UCB 60× inverted microscopy; (d-e) detection of ROS in untreated and azacytidine-treated U266 cells using the Image-iT LIVE Reactive Oxygen Species (ROS) Kit. Cells were labeled with carboxy-H_2_DCFDA, which exhibited green fluorescence when reacted with ROS, and nuclei were stained with blue-fluorescent Hoechst 33342. **(d)** untreated U266 cells; and **(e)** azacytidine-treated cells; and **(f)** gel electrophoresis of DNA from untreated and azacytidine-treated myeloma cells.

Three myeloma cell lines U266, NCI-H929, and RPMI8226 were treated with azacytidine at different concentrations. All three cell lines showed the identical morphological changes upon azacytidine treatment. FACS analysis showed that the percentage of necrotic cells was 22% when cells were treated with 5 μM azacytidine for 24 h, increasing to 38% when treated with 80 μM azacytidine for 24 h (Figure 1). Morphological features of the dying cells were consistent with the cell necrosis. Images of cell morphology in untreated, hydrogen peroxide- and azacytidine-treated cells are shown in Figure 2(a-c), respectively. The hydrogen peroxide- and azacytidine-treated myeloma cells displayed characteristic features of necrosis, including cell swelling, translucent cytoplasm, cell membrane disruption, pyknotic nuclei, and excessive cellular debris. The DNA content of necrotic cells was analyzed by gel electrophoresis. The gel image of DNA for untreated and azacytidine-treated cells (Figure 2(f)) shows that DNA from azacytidine-treated cells exhibited a random and general cleavage pattern and produced a smear that further confirmed that azacytidine-induced cell death occurs mainly via necrosis. The above data suggests that oxidative stress may cause necrosis in azacytidine- or hydrogen peroxide–treated cells. To confirm that ROS contributes to azacytidine induced cell necrosis, an Image-iT LIVE Reactive Oxygen Species (ROS) Kit was used to detect ROS in the untreated and azacytidine-treated cells. Cells were labeled with carboxy-H_2_DCFDA, which fluoresces when oxidized by ROS, and nuclei were stained with blue-fluorescent Hoechst 33342. The azacytidine-treated cells exhibited much stronger green fluorescence (Figure 2(e)) in comparison to untreated cells (Figure 2(d)), indicating that azacytidine induced a significant increase in ROS.

### Proteomic analysis and identification of cell-bound albumin in myeloma cell necrosis

**Figure 3:**
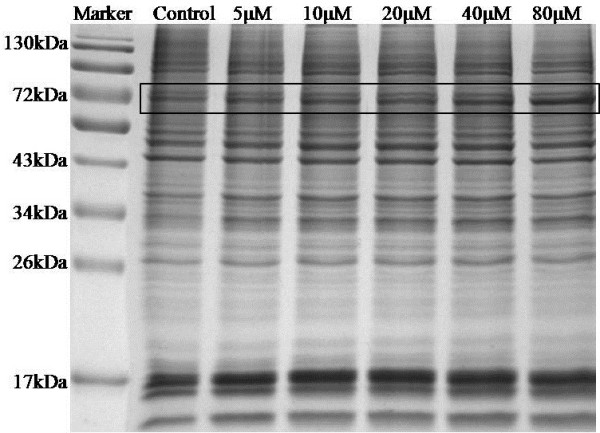
**The 1D SDS-PAGE gel image of untreated and 24 h, azacytidine-treated myeloma cells.** Lane 1, molecular weight markers; Lane 2, proteins from untreated cells; Lane 3, proteins from 5 μM azacytidine-treated cells; Lane 4, proteins from 10 μM azacytidine-treated cells; Lane 5, proteins from 20 μM azacytidine-treated cells; Lane 6, proteins from 40 μM azacytidine-treated cells; and Lane 7, proteins from 80 μM azacytidine-treated cells;. The band with the most differentially expressed proteins was marked with a square.

Next, proteomic analysis was carried out on the necrotic myeloma cells. An equal amount of proteins (30 μg) from untreated- and azacytidine-treated U266 cells were separated by 1D SDS-PAGE (Figure 3). Differentially expressed proteins were immediately visible in the gel band circled with a square. The intensity of the band at about 70 kDa showed the largest change between untreated- and azacytidine-treated samples and become more intense with higher concentrations of azacytidine. The gel band was analyzed and the major protein was identified in this band as human albumin, production of which is a hallmark of hepatocytes. Previous studies have shown that monocytes were capable of differentiation into hepatocytes under different conditions, and also that microglial cells in brain synthesized albumin [38-41]. In order to determine whether the cell-bound albumin originated from azacytidine-treated myeloma cells, a human albumin-specific antibody was used for western blot analysis. However, the antibody not only recognized the proteins in the 70 kDa band, but it also recognized bovine serum albumin (data not shown), promoting us to verify the MS/MS spectra of tryptic peptides matched those of human albumin. In fact, MS/MS spectra matched to human albumin by searching a human protein database also matched to sequences of tryptic peptides of bovine serum albumin (BSA). Furthermore, many unidentified MS/MS spectra also matched to peptide sequences of BSA. One of spectra is shown in Additional file 2: Figure S1. Indeed, searching a bovine protein database led to identification of BSA with sequence coverage of 86% of BSA sequence, demonstrating that BSA was enriched in necrotic cells. Furthermore, the intense band was no longer visible by 1D SDS-PAGE when myeloma cells were cultured in serum-free medium and treated with 100 μM azacytidine, confirming that BSA originated from cell culture medium.

Using western blot analysis, we confirmed that enrichment of cell-bound albumin was a concentration-dependent event Additional file 3: Figure S2 (a)). The intensity of the BSA band became more intense when cells were treated with higher concentrations of azacytidine for 24 h. Moreover, western blotting also shows the up-regulation of HSP60 when cells were treated with higher concentrations of azacytidine. A recent study has shown that HSP60 localizes in the tumor cell plasma membrane associated with lipid rafts [42]. In order to determine whether BSA binds to HSP60 in necrotic myeloma cells, we used an anti-BSA antibody to immune-precipitate BSA and associated proteins from untreated and azacytidine-treated myeloma cells. Immunoprecipitated proteins were separated and probed with anti-HSP60 antibodies, and western blot analysis showed that HSP60 was co-immunoprecipitated by an anti-BSA antibody (Additional file 3: Figure S2 (b)). An early study shows that the cell-bound albumin binds to peptidoglycan-, lipopolysacchride-, and lipoteichoic acid in lymphocytes and macrophages [43]. Our data indicate that HSP60 is an additional binding partner of the cell-bound albumin.

A similar protein band pattern by 1D SDS-PAGE was exhibited in Additional file: 4: Figure S3 for U266 cells treated with hydrogen peroxide, showing the characteristic band of enriched cell-bound albumin at about 70 kDa. Moreover, enrichment of BSA was observed in other myeloma cell lines RPMI8226 and NCI-H929 when they were treated with azacytidine (Additional file 5: Figure S4). It is worth mentioning that enrichment of albumin is not observed in azacytidine-treated non-hematopoietic cell lines including A549, A2780, and HUH-7 from lung, ovarian, and liver cancer, respectively. Taken together, out data suggest that enrichment of cell-bound albumin was unique to oxidative stress induced-necrosis in myeloma cell lines.

### Oxidative stress-induced over-expression of ER- and mitochondrial-specific chaperones in necrotic myeloma cells

**Figure 4:**
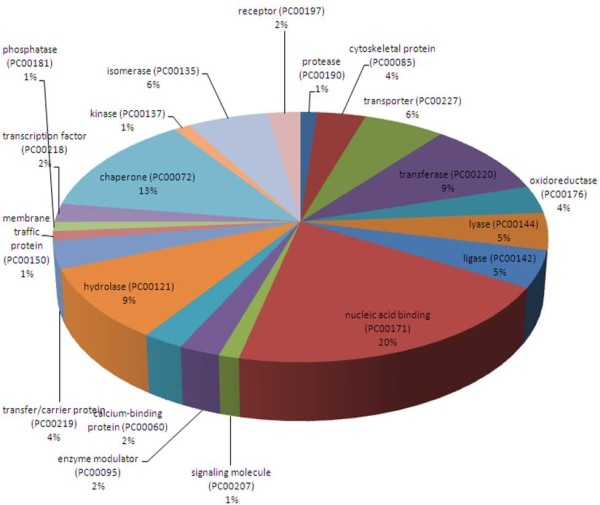
**Functional classification of differentially regulated proteins with PANTHER (**http://www.pantherdb.org**).** The numbers of proteins related with each category are shown in brackets.

We also identified changes of expression levels in other proteins in azacytidine-treated cells. Using label-free quantitation method by spectra counts and the extracted ion current, we showed that expression levels of 79 proteins were changed upon azacytidine treatment. These proteins participate in diverse cellular activities including transporting, nucleic acid binding, hydrolase activities, calcium-binding, membrane trafficking, chaperoning, and receptor binding (Figure 4), in which 17 proteins interact with nucleic acid and 11 proteins are molecular chaperones. Twenty nine up-regulated and 50 down-regulated proteins are listed in Table 1 and Table 2, respectively. Nineteen up-regulated proteins are known ER- and mitochondrialspecific proteins. ER is a multifunctional organelle that plays an essential role in protein folding, assembly and quality control of secretory and membrane proteins, disulfide bond formation, glycosylation, lipid biosynthesis, Ca^2+^ storage and signaling. Significant changes of ER- and mitochondrial proteins indicate that they are main targets of azacytidine-induced oxidative stress.

**Table 1:** **Up-regulated proteins after treatment with 100** μ**M azacytidine**

**Up regulated proteins**		**Fold changes**
IPI00784154	60 kDa heat shock protein, mitochondrial	2.4
IPI00010796	Protein disulfide-isomerase	2.0
IPI00009904	Protein disulfide-isomerase A4	1.6
IPI00020599	Calreticulin	1.7
IPI00027230	Endoplasmin	3.0
IPI00941747	Calnexin	2.6
IPI00025874	Dolichyl-diphosphooligosaccharide-protein glycosyltransferase subunit 1	2.1
IPI00453473	Histone H4	2.0
IPI00556485	RPLP0 protein	1.6
IPI00216691	Profilin-1	2.5
IPI00018206	Aspartate aminotransferase, mitochondrial	2.8
IPI00440493	ATP synthase subunit alpha, mitochondrial	2.8
IPI00020416	Tripeptidyl-peptidase 2	1.6
IPI00291006	Malate dehydrogenase, mitochondrial	3.3
IPI00339274	Histone H2A type 2-C	2.7
IPI00642982	LONP1 protein	2.3
IPI00018534	Histone H2B type 1-L	2.5
IPI00294398	Isoform 1 of Hydroxyacyl-coenzyme A dehydrogenase, mitochondrial	1.8
IPI00006482	Isoform Long of Sodium/potassium-transporting ATPase subunit alpha-1	2.1
IPI00030363	Acetyl-CoA acetyltransferase, mitochondrial	1.8
IPI00303476	ATP synthase subunit beta, mitochondrial	3.4
IPI00216308	Voltage-dependent anion-selective channel protein 1	3.3
IPI00017334	Prohibitin	2.5
IPI00001539	3-ketoacyl-CoA thiolase, mitochondrial	2.0
IPI00008529	60S acidic ribosomal protein P2	1.8
IPI00013808	Alpha-actinin-4	1.9
IPI00413611	DNA topoisomerase 1	1.8
IPI00759715	Isoform Cytoplasmic of Fumaratehydratase, mitochondrial	1.6
IPI00646304	Peptidyl-prolylcis-trans isomerase B	1.7

**Table 2:** **Down-regulated proteins after treatment with 100** μ**M azacytidine**

**Down regulated proteins**		**Fold changes**
IPI00003865	Isoform 1 of Heat shock cognate 71 kDa protein	0.5
IPI00414676	Heat shock protein HSP 90-beta	0.7
IPI00784295	Isoform 1 of Heat shock protein HSP 90-alpha	0.6
IPI00220740	Isoform 2 of Nucleophosmin	0.2
IPI00018465	T-complex protein 1 subunit eta	0.7
IPI00179330	Ubiquitin-40S ribosomal protein S27a	0.4
IPI00985384	ATP-dependent RNA helicase DDX3X isoform 3	0.2
IPI00027547	Dermcidin	0.2
IPI00376143	DNA replication licensing factor MCM7 isoform 2	0.3
IPI00786995	DNA-dependent protein kinase catalytic subunit-like	0.2
IPI00002966	Heat shock 70 kDa protein 4	0.4
IPI00027107	elongation factor Tu, mitochondrial precursor	0.4
IPI00026781	Fatty acid synthase	0.4
IPI00015018	Inorganic pyrophosphatase	0.5
IPI00008557	Insulin-like growth factor 2 mRNA-binding protein 1	0.4
IPI00000816	Isoform 1 of 14-3-3 protein epsilon	0.5
IPI00658000	Isoform 1 of Insulin-like growth factor 2 mRNA-binding protein 3	0.1
IPI00455383	Isoform 2 of Clathrin heavy chain 1	0.5
IPI00883762	Isoform 2 of Cytosolic acyl coenzyme A thioester hydrolase	0.1
IPI00025273	Isoform Long of Trifunctional purine biosynthetic protein adenosine-3	0.4
IPI00216230	Lamina-associated polypeptide 2, isoform alpha	0.5
IPI00170935	Leucine-rich repeat-containing protein 47	0.1
IPI00017297	Matrin-3	0.1
IPI00016610	Poly(rC)-binding protein 1	0.5
IPI00017617	Probable ATP-dependent RNA helicase DDX5	0.5
IPI00550451	Serine/threonine-protein phosphatase PP1-alpha catalytic subunit	0.2
IPI00947285	suprabasin isoform 1 precursor	0.3
IPI00216049	Isoform 1 of Heterogeneous nuclear ribonucleoprotein K	0.1
IPI01011912	Phosphoglycerate kinase	0.5
IPI00549725	Phosphoglyceratemutase 1	0.2
IPI00645452	Tubulin, beta	0.3
IPI00179964	Isoform 1 of Polypyrimidine tract-binding protein 1	0.3
IPI00217465	Histone H1.2	0.4
IPI00783271	Leucine-rich PPR motif-containing protein, mitochondrial	0.5
IPI00219365	Moesin	0.6
IPI00396485	Elongation factor 1-alpha 1	0.4
IPI00419258	High mobility group protein B1	0.3
IPI00031812	Nuclease-sensitive element-binding protein 1	0.3
IPI00465439	Fructose-bisphosphatealdolase A	0.5
IPI00217223	Multifunctional protein ADE2	0.4
IPI00550363	Transgelin-2	0.5
IPI00917777	116 kDa U5 small nuclear ribonucleoprotein component isoform b	0.5
IPI00100160	Isoform 1 of Cullin-associated NEDD8-dissociated protein 1	0.5
IPI00793443	Isoform 1 of Importin-5	0.4
IPI00449049	Poly [ADP-ribose] polymerase 1	0.6
IPI00007797	Fatty acid-binding protein, epidermal	0.4
IPI00398625	Hornerin	0.4
IPI00843975	Ezrin	0.6
IPI00465248	Isoform alpha-enolase of Alpha-enolase	0.5
IPI00645078	Ubiquitin-like modifier-activating enzyme 1	0.4

**Figure 5:**
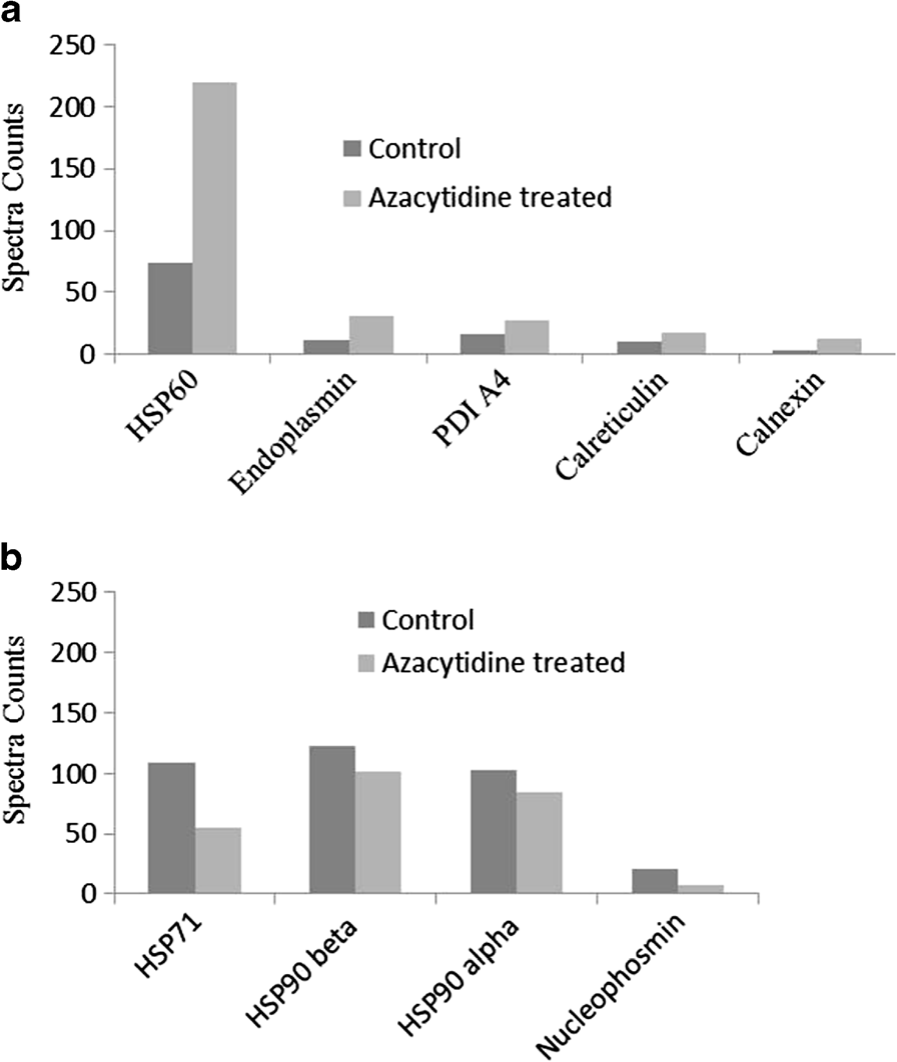
**Azacytidine induced changes in chaperones. (a)** The fold of changes of ER- and mitochondrial chaperones from the untreated and 100 μM azacytidine-treated U266 cells for 24 h; **(b)** The fold of changes of cytosolic and nuclear chaperones from untreated and 100 μM azacytidine-treated U266 cells for 24 h. The number on the y axis represents spectra counts for each identified protein.

Five known mitochondrial and ER-chaperones HSP60, protein disulfide isomerase (PDI), endoplasmin, calreticulin, and calnexin were all upregulated in U266 cells upon azacytidine treatment as indicated by changes of spectra counts (Figure 5(a)), showing that expression level of HSP60 has three-fold increase in necrotic cells. Up-regulation of HSP60 was also confirmed by western blotting (Additional file 3: Figure S2 (a)). Protein abundance index (PAI) was calculated based on the number of observed peptides per protein normalized by the theoretical number of peptides (Additional file 6: Table S2), showing that the relative concentration of HSP60 is 2 times higher than those of other four chaperones. It has been shown that HSP60 is essential in the synthesis and transportation of essential mitochondrial proteins [44] and that HSP60 is up-regulated to protect cellular survival under toxic or stressful circumstances [45-47]. Our data demonstrates that expression level of HSP60 is highly up-regulated under oxidative stress, suggesting HSP60 may serve as a new target in myeloma treatment.

PDI catalyzes formation and breakage of disulfide bonds for proteins to achieve their fully folded state and aids wrongly folded proteins to reach a correctly folded state as a chaperone [48,49]. Endoplasmin chaperone functions in the processing and transport of secreted proteins in ER and possesses the ATPase activity and calcium-binding property. Calreticulin is a lectin-like, calcium binding ER-specific chaperone that binds to misfolded proteins and prevents them from being exported from the ER to the Golgi apparatus. Overexpression of calreticulin in many cancer cells promotes macrophages to engulf hazardous cancerous cells [50]. Azacytidine also induces up-regulation of calnexin (CNX), whose main function is to assist protein folding and quality control. Calreticulin, calnexin, and ERp57 constitute the calreticulin/calnexin cycle functioning in the quality control of transmembrane and secreted glycoproteins in ER. Up-regulation of ER-specific chaperones indicates that oxidative stress activates intracellular signal transduction pathways and induces the transcriptional upregulation of genes to enhance the ER protein-folding capacity and quality control. Azacytidine induced up-regulation of mitochondrial and ER-chaperones were also observed in other treated myeloma cells as quantified by TMT labeling (Additional file 7: Table S3).

### Necrosis-induced release of HSP71 and HSP90 into the cell medium

**Figure 6:**
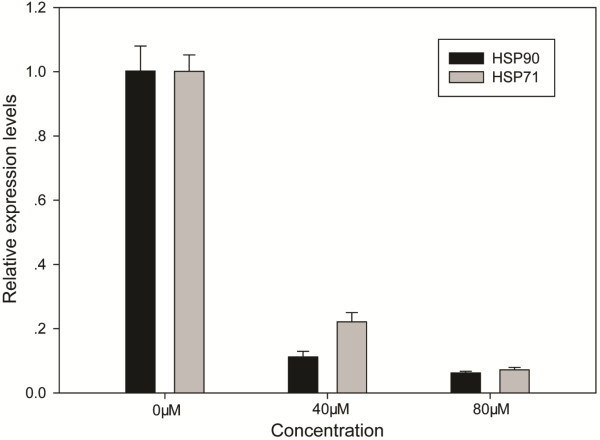
qPCR analysis of HSP90 and HSP71 from untreated cells and cells treated with 40 μM and 80 μM azacytidine for 24 h.

Expression levels in 50 proteins were down-regulated upon azacytidine treatment including 22 nucleus-specific proteins and 21 nucleotide binding proteins. Five chaperones were down-regulated including chaperonin containing TCP1 subunit 7 (eta), HSP71, HSP90 alpha (cytosolic), HSP90 beta, and nucleophosmin 2 (Figure 5(b)). To confirm azacytidine-induced down-regulation of HSP90 and HSP71, qPCR analysis was carried out, and showed that the expression of HSP90 and HSP71 at the mRNA level is down-regulated after azacytidine treatment (Figure 6). HSP71 is the major chaperone involving in protein folding and protects proteins against aggregation in cytosol. Extensive studies have shown that HSP71 is detected outside cells, where it is thought to activate the immune system [51-53]. In order to determine whether necrotic myeloma cells release HSP71 into cell medium, we analyzed proteins in culture medium of untreated and azacytidine-treated cells. Using TMT labeling, we found that relative concentrations of HSP71 and HSP90 were two times higher in medium of necrotic cells than those of untreated cells (Additional file 8: Figure S5), demonstrating enhanced release of HSP71 and HSP90 from necrotic cells into the cell culture medium. HSP71 does not have a consensus sequence for secretion, and the mechanism for translocation of this protein across membranes may involve in the binding of HSP71 with the plasma membrane before release into the extracellular environment. Chaperonin containing TCP-1 is a cytoplasmic protein and plays an important role in folding of alpha and beta tubulin. The expression level of nucleophosmin has the largest decrease upon azacytidine-treatment Figure 5(b). Nucleophosmin 2 is a histone chaperone located in the nucleolus, but it can be translocated to the nucleoplasm in response to serum starvation or drug treatment. Other down-regulated proteins participated in diverse activities such as nucleotide-binding, metabolic processes, and RNA binding (Table 2). Azacytidine is an inhibitor for DNA methyltransferase. We did not determine azacytidine-induced changes in DNA methylation in current study. We hypothesize that changes in gene expressions are attributable to both azacytidine induced epigenetic changes and ROS signaling. Great efforts are still needed to determine molecule mechanisms of azacytidine induced necrosis in myeloma cells.

## Conclusions

Taken together, our results show that azacytidine and hydrogen peroxide induce necrosis in myeloma cells through oxidative stress, resulting in enrichment of cell-bound albumin and up-regulation of ER- and mitochondrial-specific chaperones and suggesting that mitochondria and ER are major targets of ROS. Expression levels of HSP90 and HSP71 are down-regulated in azacytidine-treated cells, concomitant with enhanced release of these cytosolic chaperones into the cell culture medium. Inhibition of HSP60 may be a new therapeutic approach for myeloma treatment.

## Competing interests

We hereby declare that we have no financial or non-financial competing interest.

## Authors’ contributions

QTW and HTD designed the study and write the manuscript; EBT and HPT did all proteomic analysis; RHX and CDL participated data analysis. All authors read the draft and approved the final manuscript.

## Supplementary Material

Additional file 1: Table S1 Primers used for qPCR analysis in this work. Click here for file

Additional file 2: Figure S1 The MS/MS spectrum of a doubly charged peptide ion at m/z 863 for MH_2_^2+^ corresponding to the mass of the peptide MPCTEDYLSLILNR) from Bovine serum albumin,with four amino acids difference from human serum albumin. Click here for file

Additional file 3: Figure S2 Western blot analysis of BSA and HSP60 in azacytidine treated cells. (a) Western blot analysis of HSP60 and BSA from untreated and U266 cells treated with 20 μM, 40 μM, and 80 μM azacytidine for 24 h. Lane 1, before treatment; Lane 2, 20 μM; Lane 3, 40 μM; and Lane 4, and 80 μM. (b) Western blot analysis of HSP60 after anti-BSA antibody-immunoprecipitation. Click here for file

Additional file 4: Figure S3 The 1D SDS-PAGE gel image of proteins from untreated and H_2_O_2_-treated U266 cells. Lane 1, molecular weight markers; Lane 2, proteins from untreated cells; Lane 3, proteins from 2.5 mM H_2_O_2_-treated U266 cells; Lane 4, proteins from 5 mM H_2_O_2_-treated cells. The band with differentially expressed proteins was marked with a square. Click here for file

Additional file 5: Figure S4 The 1D SDS-PAGE gel image of proteins from untreated and azacytidine-treated RPMI8226 and NCI-H929 cells. Lane 1, molecular weight markers; Lane 2, proteins from untreated cells; Lane 3, proteins from 80 μM azacytidine-treated RPMI8226 cells; Lane 4, molecular weight markers; Lane 5, proteins from untreated NCI-H929 cells; Lane 6, proteins from 80 μM azacytidine-treated NCI-H929 cells. The band with differentially expressed proteins was marked with a square. Click here for file

Additional file 6: Table S2 Estimated protein abundance index (PAI) of HSP60, PDI, endoplasmin, calreticulin, and calnexin in untreated and 24 h, 100 μM azacytidine-treated U266 cells. Click here for file

Additional file 7: Table S3 Relative concentration ratios of selected proteins in untreated and azacytidine-treated RPMI8226 and NCI-H929 cells as determined by TMT-labeling, respectively. Click here for file

Additional file 8: Figure S5 MS/MS spectra of TMT-labeled peptides of HSP71 and HSP90. (a) The MS/MS spectrum of a doubly charged ion at m/z 739.89 for MH_2_^2+^ corresponding to the mass of the peptide TMT-labeled FEELNADLFR. The labeled peaks correspond to masses of y and b ions of the modified peptide; (b) The MS/MS spectrum of a triply charged ion at m/z 465.25 for MH_3_^3+^ corresponding to the mass of the TMT-labeled peptide LGIHEDSQNR. The labeled peaks correspond to masses of y and b ions of the modified peptide. Figure inserts show peaks of TMT reporter ions of two labeled peptides. Click here for file
