# Supplementary material for: Azacytidine induces necrosis of multiple myeloma cells through oxidative stress
Source: Proteome Sci. 2013 Jun 13;11:24. doi: 10.1186/1477-5956-11-24 (PMC3718702; doi:10.1186/1477-5956-11-24)
Supplement: Additional file 1: Table S1 — Primers used for qPCR analysis in this work. [file 1477-5956-11-24-S1.docx]

Supplementary table 1: Primers used in qPCR

| Gene name | Sequence(5'-3') |
| --- | --- |
| Human18SrRNA | forward primer GTAACCCGTTGAACCCCATT |
|  | reverse primer CCATCCAATCGGTAGTAGCG |
| HSP90AA1 | forward primer AGGAGGTTGAGACGTTCGC |
|  | reverse primer AGAGTTCGATCTTGTTTGTTCGG |
| HSP71 | forward primer ACTCCAAGCTATGTCGCCTTT |
|  | reverse primer TGGCATCAAAAACTGTGTTGGT |
