# Supplementary material for: Azacytidine induces necrosis of multiple myeloma cells through oxidative stress
Source: Proteome Sci. 2013 Jun 13;11:24. doi: 10.1186/1477-5956-11-24 (PMC3718702; doi:10.1186/1477-5956-11-24)
Supplement: Additional file 2: Figure S1 — The MS/MS spectrum of a doubly charged peptide ion at m/z 863 for MH22+ corresponding to the mass of the peptide MPCTEDYLSLILNR) from Bovine serum albumin,with four amino acids difference from human serum albumin. [file 1477-5956-11-24-S2.pdf]

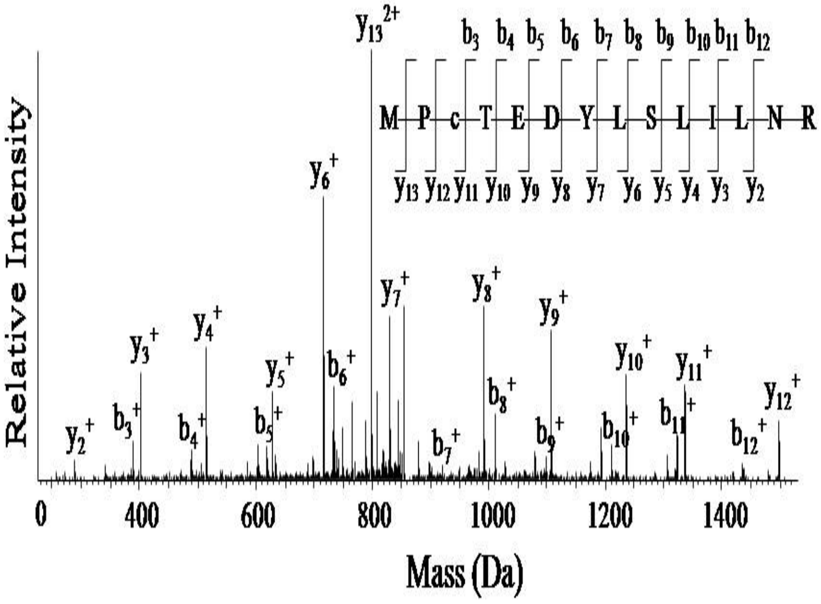


Supplementary Figures 1. The MS/MS spectrum of a doubly charged peptide ion at m/z 863 for $MH_2^{2+}$ corresponding to the mass of the peptide MPCTEDYLSLILNR) from Bovine serum albumin， with four amino acids difference from human serum albumin.
