# Supplementary material for: Azacytidine induces necrosis of multiple myeloma cells through oxidative stress
Source: Proteome Sci. 2013 Jun 13;11:24. doi: 10.1186/1477-5956-11-24 (PMC3718702; doi:10.1186/1477-5956-11-24)
Supplement: Additional file 3: Figure S2 — Western blot analysis of BSA and HSP60 in azacytidine treated cells. (a) Western blot analysis of HSP60 and BSA from untreated and U266 cells treated with 20 μM, 40 μM, and 80 μM azacytidine for 24 h. Lane 1, before treatment; Lane 2, 20 μM; Lane 3, 40 μM; and Lane 4, and 80 μM. (b) Western blot analysis of HSP60 after anti-BSA antibody-immunoprecipitation. [file 1477-5956-11-24-S3.pdf]

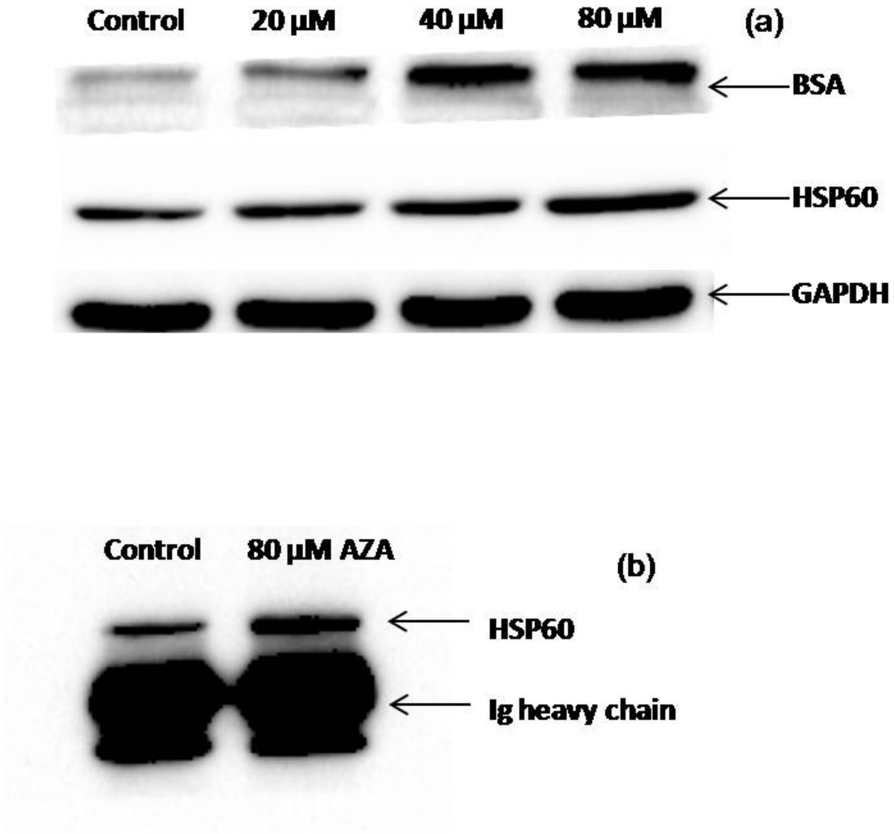

Supplementary Figure 2. Western blot analysis of BSA and HSP60 in azacytidine treated cells. (a) Western blot analysis of HSP60 and BSA from untreated and U266 cells treated with 20 µM, 40 µM, and 80 µM azacytidine for 24 h. Lane 1, before treatment; Lane 2, 20 µM; Lane 3, 40 µM; and Lane 4, and 80 µM. (b) Western blot analysis of HSP60 after anti-BSA antibody-immunoprecipitation.
