# Supplementary material for: Azacytidine induces necrosis of multiple myeloma cells through oxidative stress
Source: Proteome Sci. 2013 Jun 13;11:24. doi: 10.1186/1477-5956-11-24 (PMC3718702; doi:10.1186/1477-5956-11-24)
Supplement: Additional file 4: Figure S3 — The 1D SDS-PAGE gel image of proteins from untreated and H2O2-treated U266 cells. Lane 1, molecular weight markers; Lane 2, proteins from untreated cells; Lane 3, proteins from 2.5 mM H2O2-treated U266 cells; Lane 4, proteins from 5 mM H2O2-treated cells. The band with differentially expressed proteins was marked with a square. [file 1477-5956-11-24-S4.pdf]

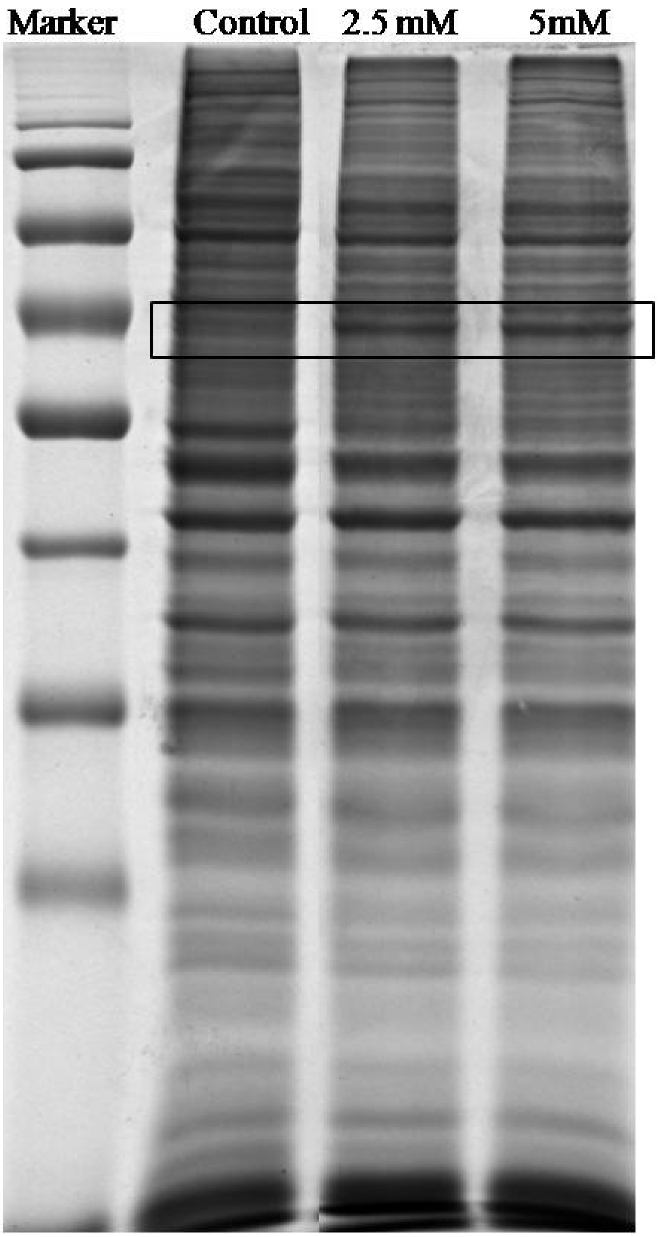

Supplementary Figures 3. The 1D SDS-PAGE gel image of proteins from untreated and H_2O_2-treated U266 cells. Lane 1, molecular weight markers; Lane 2, proteins from untreated cells; Lane 3, proteins from 2.5 mM H_2O_2-treated U266 cells; Lane 4, proteins from 5 mM H_2O_2-treated cells. The band with differentially expressed proteins was marked with a square.
