# Supplementary material for: Azacytidine induces necrosis of multiple myeloma cells through oxidative stress
Source: Proteome Sci. 2013 Jun 13;11:24. doi: 10.1186/1477-5956-11-24 (PMC3718702; doi:10.1186/1477-5956-11-24)
Supplement: Additional file 5: Figure S4 — The 1D SDS-PAGE gel image of proteins from untreated and azacytidine-treated RPMI8226 and NCI-H929 cells. Lane 1, molecular weight markers; Lane 2, proteins from untreated cells; Lane 3, proteins from 80 μM azacytidine-treated RPMI8226 cells; Lane 4, molecular weight markers; Lane 5, proteins from untreated NCI-H929 cells; Lane 6, proteins from 80 μM azacytidine-treated NCI-H929 cells. The band with differentially expressed proteins was marked with a square. [file 1477-5956-11-24-S5.pdf]

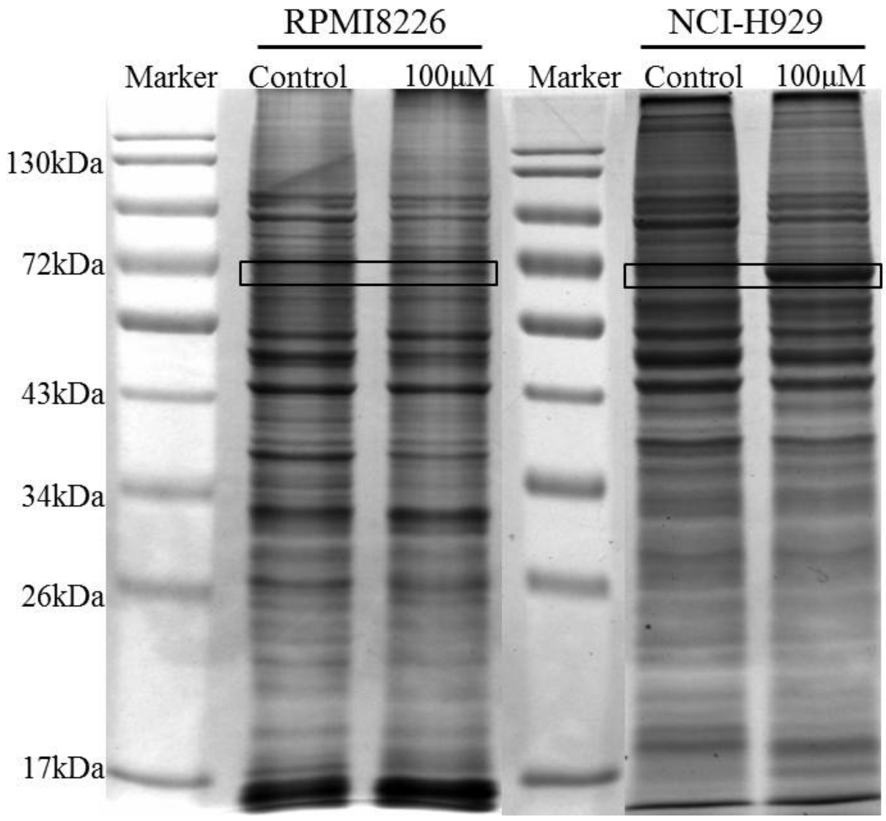

Supplementary Figure 4. The 1D SDS-PAGE gel image of proteins from untreated and azacytidine-treated RPMI8226 and NCI-H929 cells. Lane 1, molecular weight markers; Lane 2, proteins from untreated cells; Lane 3, proteins from 80 µM azacytidine-treated RPMI8226 cells; Lane 4, molecular weight markers; Lane 5, proteins from untreated NCI-H929 cells; Lane 6, proteins from 80 µM azacytidine-treated NCI-H929 cells. The band with differentially expressed proteins was marked with a square.
