# Supplementary figures and images for: Azacytidine induces necrosis of multiple myeloma cells through oxidative stress
Source: Proteome Sci. 2013 Jun 13;11:24. doi: 10.1186/1477-5956-11-24 (PMC3718702; doi:10.1186/1477-5956-11-24)

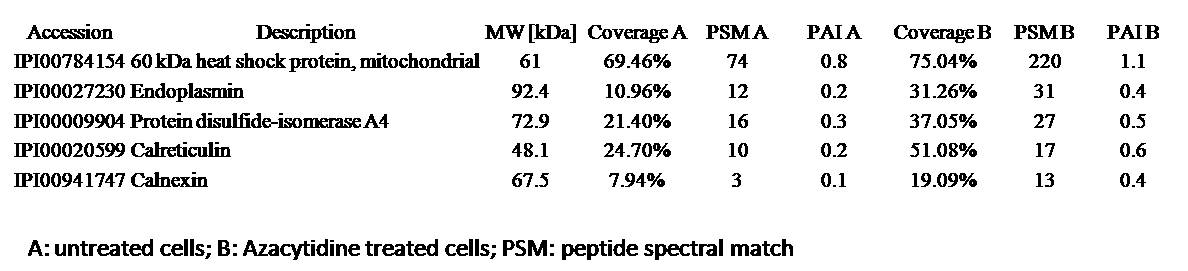

Supplement: Additional file 6: Table S2 — Estimated protein abundance index (PAI) of HSP60, PDI, endoplasmin, calreticulin, and calnexin in untreated and 24 h, 100 μM azacytidine-treated U266 cells. [file 1477-5956-11-24-S6.jpeg]

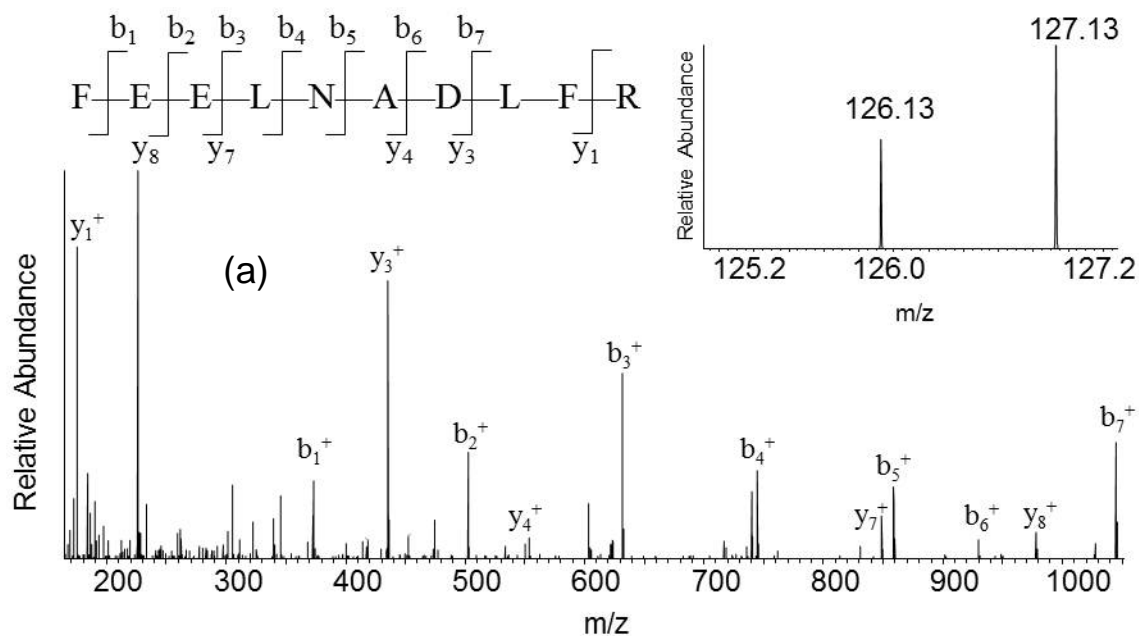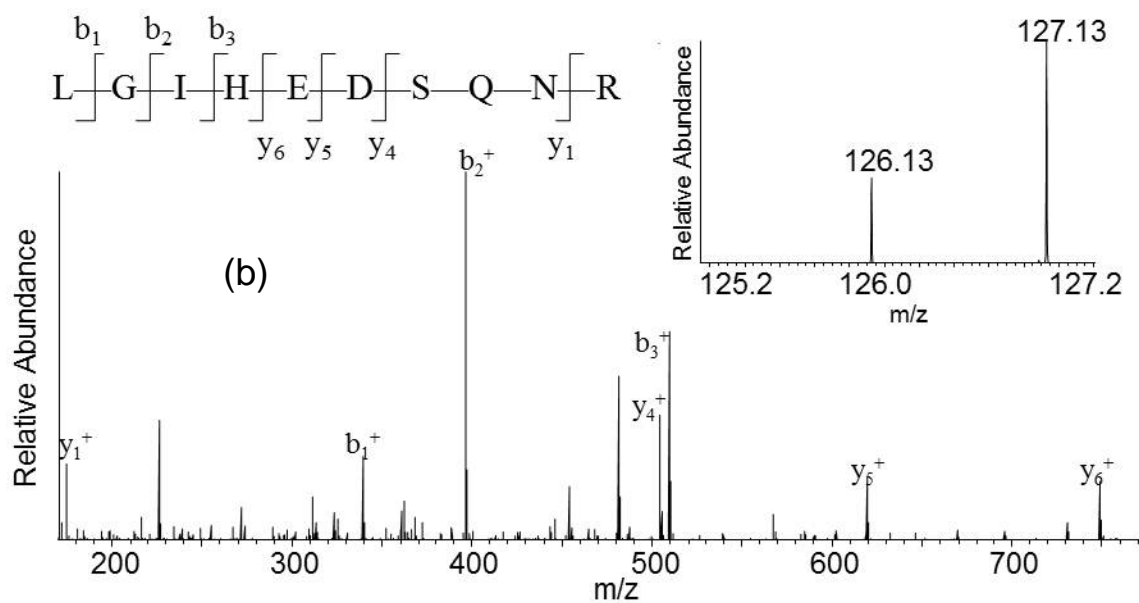

Supplement: Additional file 8: Figure S5 — MS/MS spectra of TMT-labeled peptides of HSP71 and HSP90. (a) The MS/MS spectrum of a doubly charged ion at m/z 739.89 for MH22+ corresponding to the mass of the peptide TMT-labeled FEELNADLFR. The labeled peaks correspond to masses of y and b ions of the modified peptide; (b) The MS/MS spectrum of a triply charged ion at m/z 465.25 for MH33+ corresponding to the mass of the TMT-labeled peptide LGIHEDSQNR. The labeled peaks correspond to masses of y and b ions of the modified peptide. Figure inserts show peaks of TMT reporter ions of two labeled peptides. [file 1477-5956-11-24-S8.pdf]
