# Supplementary material for: Azacytidine induces necrosis of multiple myeloma cells through oxidative stress
Source: Proteome Sci. 2013 Jun 13;11:24. doi: 10.1186/1477-5956-11-24 (PMC3718702; doi:10.1186/1477-5956-11-24)
Supplement: Additional file 7: Table S3 — Relative concentration ratios of selected proteins in untreated and azacytidine-treated RPMI8226 and NCI-H929 cells as determined by TMT-labeling, respectively. [file 1477-5956-11-24-S7.docx]

| Accession | Description | TMT ratio(Azacytidine/Control) | |
| --- | --- | --- | --- |
|  |  | RPMI8226 | NCI-H929 |
| IPI00784154 | 60 kDa heat shock protein, mitochondrial | 1.8 | 2.2 |
| IPI00027230 | Endoplasmin | 1.7 | 1.4 |
| IPI00941747 | Calnexin | 2.0 | 1.8 |
| IPI00009904 | Protein disulfide-isomerase A4 | 1.8 | 1.7 |
| IPI00003865 | Isoform 1 of Heat shock cognate 71 kDa protein | 0.7 | 0.5 |
| IPI00414676 | Heat shock protein HSP 90-beta | 0.6 | 0.5 |
| IPI00784295 | Isoform 1 of Heat shock protein HSP 90-alpha | 0.5 | 0.5 |
|  | Bovine serum albumin | 2.0 | 1.8 |
